# A neurodegenerative perspective on mitochondrial optic neuropathies

**DOI:** 10.1007/s00401-016-1625-2

**Published:** 2016-09-30

**Authors:** Patrick Yu-Wai-Man, Marcela Votruba, Florence Burté, Chiara La Morgia, Piero Barboni, Valerio Carelli

**Affiliations:** 1 Wellcome Trust Centre for Mitochondrial Research, Institute of Genetic Medicine, Newcastle University, Newcastle upon Tyne, NE1 3BZ UK; 2 Newcastle Eye Centre, Royal Victoria Infirmary, Newcastle upon Tyne, NE1 4LP UK; 3 NIHR Biomedical Research Centre at Moorfields Eye Hospital and UCL Institute of Ophthalmology, London, EC1V 2PD UK; 4 School of Optometry and Vision Sciences, Cardiff University, Cardiff, UK; 5 Cardiff Eye Unit, University Hospital of Wales, Cardiff, UK; 6 IRCCS Institute of Neurological Sciences of Bologna, Bellaria Hospital, Bologna, Italy; 7 Unit of Neurology, Department of Biomedical and Neuromotor Sciences (DIBINEM), University of Bologna, Bologna, Italy; 8 Studio Oculistico d’Azeglio, Bologna, Italy; 9 San Raffaele Scientific Institute, Milan, Italy

**Keywords:** Dominant optic atrophy, Leber hereditary optic neuropathy, Mitochondrial diseases, Neurodegenerative diseases, OPA1, Retinal ganglion cell

## Abstract

Mitochondrial optic neuropathies constitute an important cause of chronic visual morbidity and registrable blindness in both the paediatric and adult population. It is a genetically heterogeneous group of disorders caused by both mitochondrial DNA (mtDNA) mutations and a growing list of nuclear genetic defects that invariably affect a critical component of the mitochondrial machinery. The two classical paradigms are Leber hereditary optic neuropathy (LHON), which is a primary mtDNA disorder, and autosomal dominant optic atrophy (DOA) secondary to pathogenic mutations within the nuclear gene *OPA1* that encodes for a mitochondrial inner membrane protein. The defining neuropathological feature is the preferential loss of retinal ganglion cells (RGCs) within the inner retina but, rather strikingly, the smaller calibre RGCs that constitute the papillomacular bundle are particularly vulnerable, whereas melanopsin-containing RGCs are relatively spared. Although the majority of patients with LHON and DOA will present with isolated optic nerve involvement, some individuals will also develop additional neurological complications pointing towards a greater vulnerability of the central nervous system (CNS) in susceptible mutation carriers. These so-called “plus” phenotypes are mechanistically important as they put the loss of RGCs within the broader perspective of neuronal loss and mitochondrial dysfunction, highlighting common pathways that could be modulated to halt progressive neurodegeneration in other related CNS disorders. The management of patients with mitochondrial optic neuropathies still remains largely supportive, but the development of effective disease-modifying treatments is now within tantalising reach helped by major advances in drug discovery and delivery, and targeted genetic manipulation.

**Electronic supplementary material:**

The online version of this article (doi:10.1007/s00401-016-1625-2) contains supplementary material, which is available to authorized users.

## Introduction

Mitochondrial diseases affect at least 1 in 4300 of the population and patients can present with either isolated or multisystemic organ involvement [58]. Despite these wide phenotypic manifestations, the eye is particularly vulnerable and over half of all patients will develop significant ocular complications, in particular involvement of the optic nerve [35, 143]. Unsurprisingly, the risk of blindness ranks high on patients’ fears about the prognosis associated with their specific mitochondrial genetic defect [12, 74]. The optic nerve consists of the projecting axons from about 1.2 million retinal ganglion cells (RGCs) and the pathophysiological pathways that drive the selective loss of this highly specialised neuronal population are providing important new insights into the broader link between mitochondrial dysfunction and neurodegeneration within the central nervous system (CNS) [71]. The two classical mitochondrial optic neuropathies are Leber hereditary optic neuropathy (LHON) and autosomal dominant optic atrophy (DOA), which share overlapping clinical and pathological features, despite being caused by mitochondrial DNA (mtDNA) point mutations and a growing list of nuclear genetic defects, respectively. The defining neuropathological feature of LHON and DOA is the early loss of RGCs within the papillomacular bundle resulting in an expanding field defect, known as a scotoma, within the patient’s central vision [52]. Interestingly, a subgroup of patients can develop a syndromic form of LHON and DOA that is characterised by prominent neurological deficits in addition to optic atrophy. This review will focus on the disease mechanisms that underpin the selective vulnerability of RGCs in mitochondrial optic neuropathies, but also how these could relate to the more extensive CNS neurodegeneration observed in patients with the more severe “plus” phenotypes. Crucially for patients and their families, there are currently limited treatment options and a number of strategies are being considered to prevent or halt neuronal loss in this group of disorders, all of which will need to be rigorously assessed for safety and efficacy.

## Leber hereditary optic neuropathy

LHON is a primary mitochondrial genetic disorder that affects approximately 1 in 30,000 people in the population and three point mutations within the mitochondrial genome account for over 90 % of cases, namely m.3460G>A (*MTND1*), m.11778G>A (*MTND4*) and m.14484T>C (*MTND6*) [87]. These three primary mtDNA mutations all affect complex I subunits and the disturbed flux of electrons along the mitochondrial respiratory chain results in impaired oxidative phosphorylation (OXPHOS) and increased levels of reactive oxygen species (ROS) [32]. LHON is the classical paradigm of a mitochondrial optic neuropathy and the preferential loss of retinal ganglion cells (RGCs) raises intriguing questions relating to tissue specificity and the secondary modulatory factors that dictate disease expression. The m.11778G>A LHON mutation was the first mtDNA variant linked with human disease and despite three decades of intensive research, there are still several unsolved mysteries, in particular the marked incomplete penetrance and the sex bias associated with this mitochondrial disorder [134]. Penetrance can vary widely between families or even within different branches of the same family, but as a rule of thumb, the penetrance for a male LHON carrier is ~50 % compared with ~10 % for a female LHON carrier [35, 143]. All the evidence so far points towards LHON being a complex disease that is determined by additional mitochondrial and nuclear genetic risk factors as well as environmental triggers such as smoking, which is associated with significantly increased risk of visual loss [66, 75, 88]. Oestrogens could also have a neuroprotective effect on RGCs and this feature could account, at least partly, for the relative protection of female LHON carriers against visual loss [55].

**Fig. 1:**
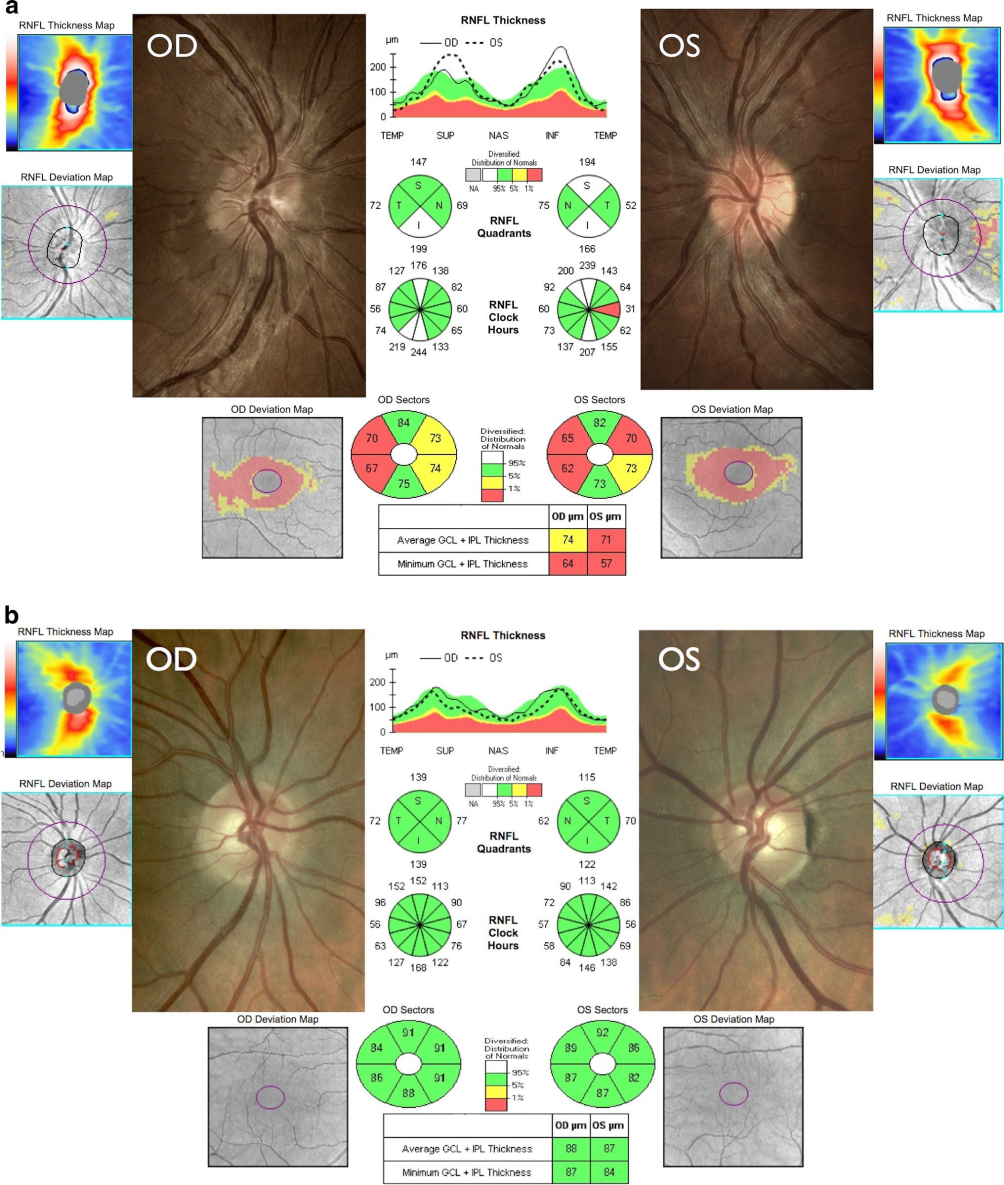
Colour fundus and OCT findings in a patient with acute LHON compared with a healthy subject. **a** An 18-year-old man harbouring the m.3460G>A mtDNA mutation who was in the acute stage of LHON with onset of visual loss of 1 and 2 months in the right (OD) and left eyes (OS), respectively. There are telangiectatic microangiopathy and swelling of the peripapillary retinal nerve fibre layer (RNFL) mainly involving the superior and inferior quadrants (ONH cube 200 × 200 protocol, Cirrus HD-OCT, Carl Zeiss Meditec). The left optic disc is showing early temporal pallor consistent with the RNFL thinning observed on OCT. The bottom panel shows macular ganglion cell layer thinning that is more evident in the left eye (Macular Cube 512 × 128 protocol). **b** Comparative images for a healthy subject. Please refer to the Supplementary Appendix for a more detailed explanation of the OCT measurements and their anatomical correlates

Patients with LHON typically present with bilateral subacute loss of central vision and the time course is defined by an expanding dense scotoma and a period of worsening visual acuities until a nadir is reached. In unilateral cases, the fellow eye usually converts within 3-6 months after first disease onset (Fig. 1). Spontaneous visual recovery can occur, usually in the first year, but the prognosis remains guarded with most patients remaining within the legal criteria for blind registration [97]. Limited post-mortem studies of optic nerves from patients with LHON confirm that the neuropathology is limited to the RGC layer [82, 116]. The rapid loss of RGCs in the acute phase of the disease is thought to be mediated via apoptotic pathways, but further work is needed to determine whether the primary site of the pathology lies within the cell body or the proximal unmyelinated axonal segments. The acute phase of LHON is characterised by the early loss of RGCs within the papillomacular bundle and this interesting pathological feature has been ascribed to their smaller than average axonal calibre [118]. The basis for this anatomical vulnerability has been modelled using a mathematical equation that integrates the surface area (energy demand) and volume (energy supply) ratio of the RGC axons, in relation to the amount of ATP needed to maintain axonal conduction [101]. It must be emphasised that there is no absolute experimental proof, but the greater surface area/volume ratio in smaller fibres is thought to result in a more limited mitochondrial bioenergetic reserve capacity and proportionally more ROS production compared with larger fibres [83].

## LHON plus phenotypes

Although the neuropathological hallmark of LHON is dominated by the loss of RGCs within the inner retina, a subgroup of patients will develop extraocular features as part of a more severe LHON “plus” syndromic phenotype [98]. It can be difficult to conclusively ascertain the link between a specific neurological feature and a LHON mtDNA mutation, but multiple independent case reports of patients developing similar syndromic phenotypes on the background of different pathogenic LHON mutations lend support to a true causal relationship, rather than mere coincidence. Dystonia and myoclonus are two movement disorders that have been consistently associated with the three primary LHON mutations and the development of these extraocular features clearly indicates the potential for a more generalised neurodegenerative process among at-risk LHON carriers [38, 92]. Rarer pathogenic mtDNA variants (m.4160T>C, m.11696A>G and/or m.14596T>A, and m.14459G>A) in isolated families from Holland, Australia and North America have previously been linked with particularly severe LHON “plus” phenotypes complicated by spastic dystonia, ataxia, juvenile-onset encephalopathy, and psychiatric disturbances [35, 143]. Two mtDNA point mutations, m.3376G>A and m.3697G>A, with a pronounced inhibitory effect on complex I activity have also been identified in patients with an LHON-like optic neuropathy and features of the MELAS (mitochondrial encephalomyopathy, lactic acidosis, and stroke-like episodes) syndrome [24, 124].

**Fig. 2:**
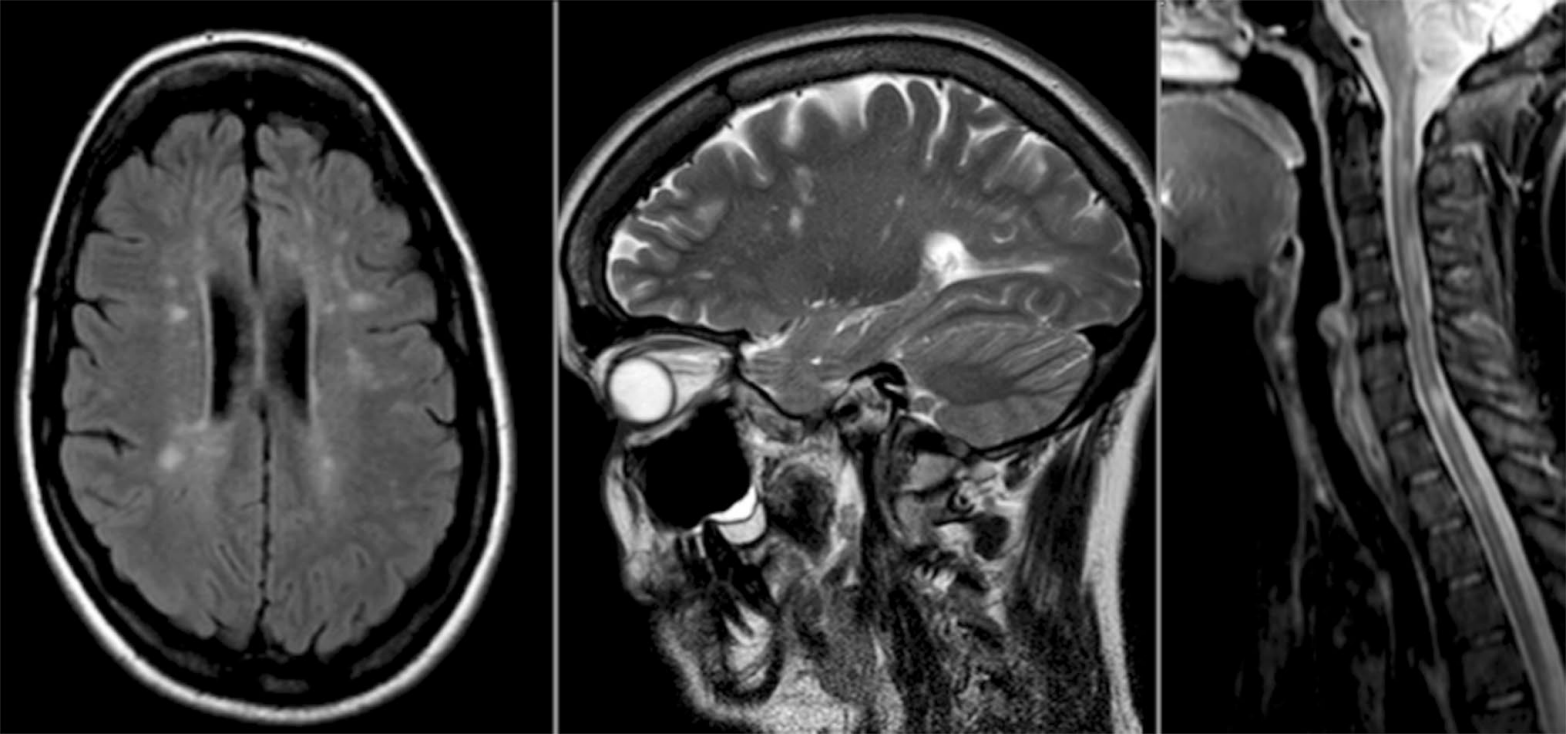
LHON-MS overlap syndrome. T2 MRI images from a 27-year-old woman with episodes of recurrent optic neuritis over a 10-year period associated with partial visual recovery. There are disseminated high-signal changes within the white matter regions of the brain and cervical spinal cord that are consistent with demyelination [105]

The overlap between LHON and a multiple sclerosis (MS)-like illness (Harding disease) is a fascinating association that highlights a potential final common pathway linking mitochondrial dysfunction with neuronal loss and disease progression in acquired demyelinating disorders affecting the central nervous system (CNS). Although Harding disease was initially described in a group of 8 female LHON carriers harbouring the m.11778G>A mutation, this striking combination of visual loss and more generalised CNS demyelination can affect both sexes, albeit with a slight female bias, and it has been reported with the m.3460G>A and m.14484T>C mutations as well (Fig. 2) [63, 81, 100, 104]. The pattern of visual loss in LHON-MS appears distinct from classical LHON and demyelinating optic neuritis, being marked by recurrent episodes of visual loss that can be associated with ocular pain, but with incomplete visual recovery and progression to registrable blindness in half of all patients [104]. Although there is conflicting evidence in the literature, on balance, it appears that a primary LHON mutation does not increase the risk of developing MS per se, but rather it modulates or potentiates the underlying neurodegenerative process in an individual who already has a pre-existing predisposition to developing clinically definite MS. As LHON-MS represents a relatively rare subgroup, there is no robust evidence base to guide treatment, but given the poor visual prognosis and the accumulating visual deficits associated with each attack, early disease-modifying therapy to prevent relapses and protect the optic nerve seems a pragmatic management approach.

Neuromyelitis optica (NMO) is a relatively rare form of CNS demyelinating disease among White Caucasians with a prevalence that is 50–100 times lower than that of MS [68]. The inflammatory process has a marked predilection for the optic nerves and the spinal cord and the neuropathology is closely linked with a specific autoantibody directed against aquaporin-4 (AQP4), which is the major water channel in the CNS [103, 138]. After decades of debate, clinicopathological studies have revealed striking differences between NMO and prototypic MS firmly establishing the two of them as distinct disease entities [23, 95, 113]. It is, therefore, interesting that in addition to Harding disease, patients with LHON have been described who developed a prominent spinal cord syndrome mimicking the radiological and pathological features of NMO [91, 122]. Additional research is clearly needed to further explore the interplay between a pathogenic LHON mutation, mitochondrial dysfunction and concurrent white matter CNS demyelinating disease [30, 85].

## Autosomal dominant optic atrophy

**Fig. 3:**
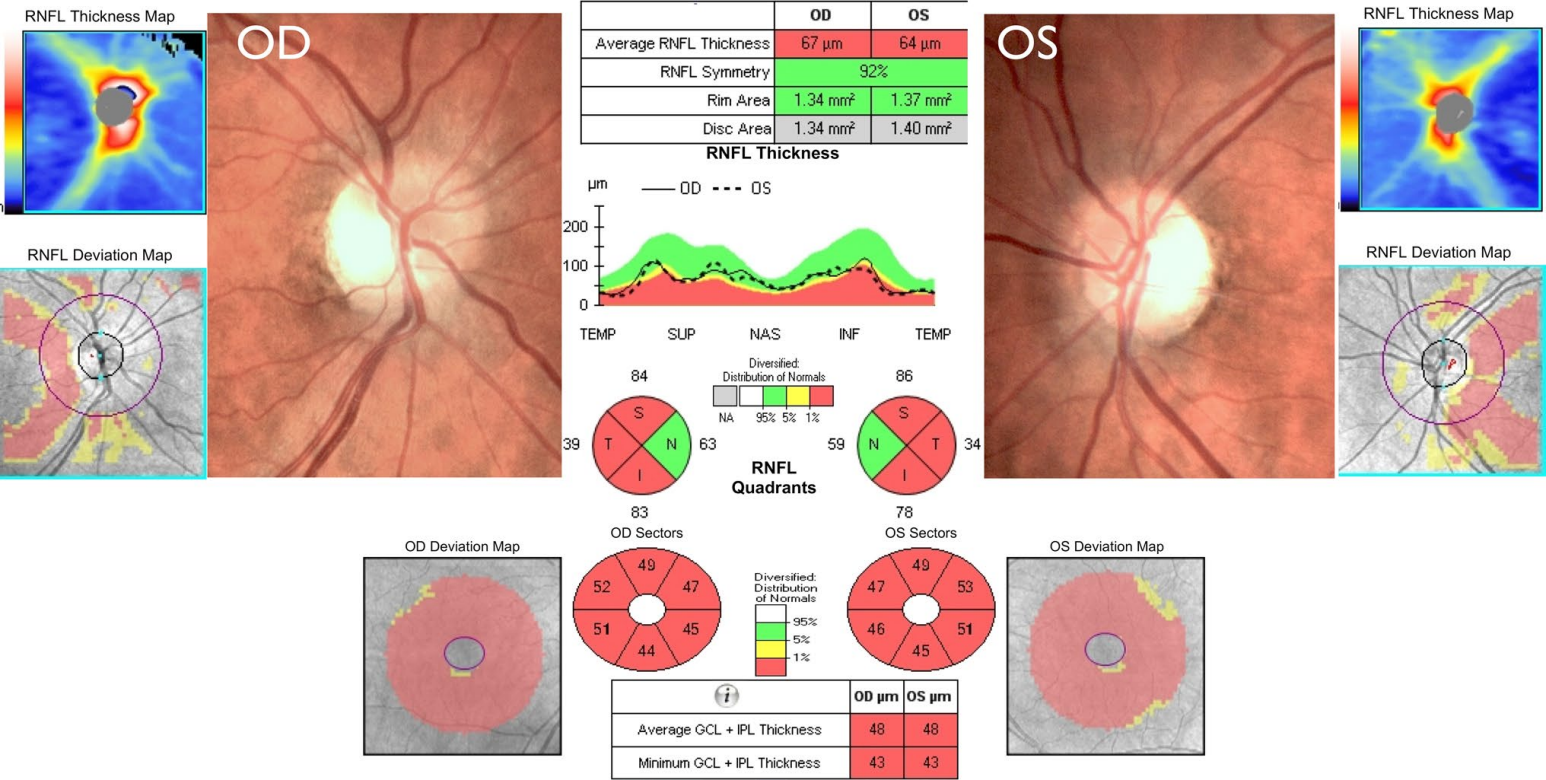
Colour fundus and OCT findings in DOA. The images were obtained from a 20-year-old woman with progressive visual loss starting in early childhood and confirmed to harbour a pathogenic *OPA1* mutation. There is prominent temporal optic disc pallor and marked RNFL thinning except in the nasal quadrant, which is relatively spared. The disc area analysis reveals small optic discs in both eyes. The bottom panel shows pronounced macular ganglion cell layer thinning in all sectors. Please refer to the Supplementary Appendix for a more detailed explanation of the OCT measurements and their anatomical correlates

In contrast to the subacute visual failure observed among young adult men in LHON, Poul Kjer described in 1959 a dominantly inherited bilateral optic neuropathy with an indolent course starting in early childhood [76]. Despite these major differences in presentation, DOA is also characterised by the marked vulnerability of RGCs within the papillomacular bundle, resulting in a central visual field defect, impaired colour vision and prominent temporal pallor of the optic disc on fundus examination (Fig. 3) [35, 143]. DOA affects at least 1 in 25,000 people in the population and it is the most common inherited optic neuropathy encountered in clinical practice [142]. The true prevalence is likely to be higher due to the marked variability in clinical expression and disease progression both within and between families. Some mutation carriers present with very poor visual function from birth with optic nerve hypoplasia, whereas others are visually asymptomatic and they are only detected as part of familial contact tracing [15, 42]. The natural history of the disease can also be highly variable with slowly progressive, sometimes step-like deterioration of visual function over time in most patients, whereas for others, visual acuity reaches a plateau in early to mid-adulthood without any further significant deterioration [41, 142]. Although the pattern of visual failure in DOA is relatively less severe compared with LHON, the majority of patients are eventually registered legally blind and they, therefore, need to be counselled appropriately.

The majority (60–70 %) of patients with DOA harbour pathogenic mutations in the nuclear gene *OPA1* that encodes for a mitochondrial inner membrane protein with multifunctional properties [4, 47]. Over 250 *OPA1* mutations have been reported and these can be grouped into two major categories depending on whether they are predicted to cause disease due to haploinsufficiency (deletions, insertions, splice site and nonsense mutations) or a possible dominant-negative mechanism (missense mutations) [50, 53]. More recently, heterozygous mutations in a number of nuclear genes have been identified in patients with DOA, including dominant *OPA3* mutations in families segregating optic atrophy and early onset cataracts [111]. The OPA3 protein is a mitochondrial outer membrane protein with pro-fission properties and the loss of RGCs has been linked to disturbed mitochondrial dynamics [28]. Interestingly, DRP1 is also a pro-fission cytosolic protein that is recruited to the mitochondrial outer membrane, and both *SPG7* and *AFG3L2* encode for mitochondrial AAA proteases that operate as oligomeric complexes to regulate the post-translational processing of OPA1 [39, 78, 135]. Rather strikingly, with the exception of *WFS1*, which encodes for an endoplasmic reticulum protein, all proteins associated with DOA are closely involved in regulating mitochondrial dynamics, pointing towards a key biological pathway essential for RGC maintenance and survival (Table 1) [28, 110]. Even with the advent of next-generation whole exome and genome sequencing, a proportion of patients with optic atrophy and a clear-cut autosomal dominant pattern of inheritance still remain genetically undefined and DOA is proving to be much more genetically heterogeneous than originally considered.

**Table 1:** Nuclear mitochondrial disorders with prominent optic nerve involvement

Inheritance	Locus	Gene	OMIM	Phenotype
Dominant	1p36.2	*MFN2*	601,152	Hereditary motor and sensory neuropathy type 6 (HMSN-6, CMT2A)
	3q28-q29	*OPA1*	165,500	Isolated optic atrophy and syndromic dominant optic atrophy (DOA plus)
	4p16.1	*WFS1*	614,296	Wolfram syndrome spectrum disorders
	12p11.21	*DRP1*	614,388	Optic atrophy and abnormal brain development
	19q13.2-q13.3	*OPA3*	165,300	Autosomal dominant optic atrophy and early-onset cataracts (ADOAC)
Recessive	3q26.33	*DNAJC19*	610,198	DCMA syndrome ± optic atrophy
	4p16.1	*WFS1*	222,300	Wolfram syndrome 1
	4q24	*CISD2*	604,928	Wolfram syndrome 2
	5q.22.1	*SLC25A46*	616,505	Optic atrophy ± peripheral neuropathy/cerebellar syndrome
	6q21	*RTN4IP1*	616,732	Optic atrophy ± cerebellar syndrome/mental retardation/epilepsy
	9q13-q21.1	*FXN*	229,300	Friedreich ataxia
	11q14.1-q21	*TMEM126A*	612,989	Optic atrophy ± auditory neuropathy
	12q24.31	*C12orf65*	615,035	Optic atrophy ± spastic paraplegia/peripheral neuropathy
	16q24.3	*SPG7*	607,259	Hereditary spastic paraplegia type 7 (HSP-7)
	19q13.2-q13.3	*OPA3*	258,501	Type III 3-methylglutaconic aciduria (Costeff syndrome)
	22q13.2	*ACO2*	616,289	Optic atrophy ± cerebellar syndrome/encephalopathy
	Xq22.1	*TIMM8A*	304,700	Mohr-Tranebjaerg syndrome ± optic atrophy

## DOA plus phenotypes

Optic atrophy is the defining feature of DOA, but a specific missense mutation within the *OPA1* gene, c.1334G >A (p.Arg455His), has been consistently associated with sensorineural deafness in a number of families [6, 144]. The clinical manifestations observed in *OPA1* disease have now expanded further to include chronic progressive external ophthalmoplegia (CPEO) and other extraocular features such as ataxia, myopathy and peripheral neuropathy [7, 13, 65]. In a large multicentre study of 104 patients with DOA plus phenotypes from 45 independent families, up to 20 % of familial *OPA1* carriers developed multisystemic neuromuscular complications and the risk was significantly increased among those harbouring missense *OPA1* mutations, consistent with a putative dominant-negative effect [144]. Rather unexpectedly, analysis of muscle biopsies obtained from this group of patients revealed multiple mtDNA deletions and the presence of high levels of cytochrome *c* oxidase (COX)-deficient muscle fibres, some of which had marked mitochondrial proliferation in the form of ragged-red fibres [125, 144]. The accumulation of mtDNA deletions is a fascinating observation that could be due to the accelerated clonal expansion of these somatic mutations to sufficiently high levels to trigger a biochemical COX defect [102, 146]. CPEO is a classical manifestation of mitochondrial disease and in keeping with the propensity of extraocular muscles to accumulate somatic mtDNA mutations at a faster rate compared with skeletal muscle, it is perhaps not surprising that about half of all patients with DOA plus phenotypes develop ptosis and ophthalmoplegia in later life [59, 145].

Similar to Harding disease in LHON, *OPA1* mutation carriers can also develop an MS-like illness where the optic atrophy occurs on the background of a more disseminated inflammatory process with neuroradiological and serological features, consistent with MS [133, 147]. It is intriguing that both LHON and DOA share this specific association and the contribution of the underlying pathogenic mtDNA or *OPA1* mutation in driving CNS neurodegeneration deserves further study. The association of optic atrophy with spastic paraplegia, resembling cases that fit the historical description of Behr syndrome, has been well described in several unrelated *OPA1* mutation carriers [26, 37, 90]. The development of this syndromic form of DOA has been ascribed to the deleterious synergistic consequences of compound heterozygous *OPA1* mutations, in particular the recurrent c.1146A>G (p.Ile382Met) missense mutation, which can occur in combination with a deep intronic mutation [25]. Remarkably, two Italian families carrying different *OPA1* missense mutations have been reported with an atypical combination of parkinsonism, dementia and CPEO, but with only subclinical optic neuropathy [34]. More recently, a compound homozygous *OPA1* mutation has been identified for the first time in two affected Jewish sisters from consanguineous parents who developed a fatal infantile encephalomyopathy with hypertrophic cardiomyopathy and optic atrophy [123]. However, instead of multiple mtDNA deletions, the muscle biopsy from one sister showed marked mtDNA depletion. The expanding clinical and genetic heterogeneity linked to DOA plus phenotypes only serves to emphasise the more global neurodegenerative impact of pathogenic *OPA1* mutations that can extend far beyond the inner retina and the anterior visual pathways. There is now a considerable body of evidence linking mitochondrial dysfunction with late-onset neurodegenerative disorders, such as Alzheimer disease and Parkinson disease, indicating a common pathological thread and a close relationship with mitochondrial structure, function and localisation [28].

A syndromic form of optic atrophy reminiscent of DOA plus can arise from dominant mutations in the *MFN2* gene, which encodes for a mitochondrial outer membrane protein [109, 115]. MFN2 coordinates mitochondrial fusion by working in close tandem with its fellow fusogenic protein, OPA1, and analogous to the observation in muscle biopsies from patients carrying *OPA1* mutations, *MFN2* mutations can also precipitate mtDNA instability with either the accumulation of multiple mtDNA deletions or mtDNA depletion. MtDNA maintenance is clearly intrinsically linked to the delicate balance between mitochondrial fusion and fission and adding further weight to this hypothesis, *SPG7* and *AFG3L2* mutations have now been reported in patients with ataxic disorders complicated by CPEO and optic atrophy, and with evidence of multiple mtDNA deletions in skeletal muscle biopsy specimens [57, 105]. Nuclear genes that are directly or indirectly involved in regulating mitochondrial dynamics and the faithful replication of the mitochondrial genome can, therefore, lead to varying degrees of CNS neurodegeneration, but with the optic nerve being invariably the most sensitive affected end organ [31].

## Other mitochondrial optic neuropathies

Autosomal recessive and X-linked optic neuropathies are relatively rare, but an increasing number of disease causing genes are being identified with wider access to molecular genetic testing and next-generation sequencing platforms (Table 1). *TMEM126A*, which encodes for a mitochondrial inner membrane protein of unclear function, was the first gene to be found in patients with non-syndromic recessive optic atrophy, although it can also occur in combination with peripheral neuropathy in some mutation carriers [62]. Other genes associated with both isolated and syndromic forms of recessive optic atrophy include: (1) *ACO2*, which encodes for an enzyme of the tricarboxylic acid cycle [93]; (2) *RTN4IP1*, which encodes for a mitochondrial outer membrane protein that interacts with the endoplasmic reticulum protein RTN4 (or NOGO) to regulate neuronal dendritic branching [9]; (3) *WFS1* (Wolfram syndrome 1), which encodes for Wolframin, a transmembrane endoplasmic reticulum protein that plays a critical role in calcium homeostasis and interorganellar cross-talk at mitochondria-associated membranes (MAMs) [28, 126]; and (4) *CISD2* (Wolfram syndrome 2), which encodes for Miner 1, a redox-active iron-sulphur cluster protein that regulates the unfolded protein response and calcium homeostasis [136]. The molecular elucidation of both forms of Wolfram syndrome has uncovered the intimate dynamic interactions between mitochondria and the endoplasmic reticulum, and how dysfunction in one compartment can detrimentally disturb the other, and vice versa.

The occurrence of visual pathway involvement has become much better appreciated in the inherited ataxias and peripheral neuropathy syndromes. Friedreich Ataxia (FRDA) is the most common form of hereditary ataxia and it is due to recessive mutations in the *FXN* gene, which encodes for a mitochondrial protein involved in the biosynthetic pathways of iron-sulphur clusters [143]. The latter are essential components of aconitase and the mitochondrial respiratory chain complexes I, II and III, and their combined dysfunction probably contributes to the development of optic neuropathy, which is now a well-recognised feature of FRDA [51]. Costeff Syndrome or type III 3-methylglutaconic aciduria (MGA) is a rare neurodegenerative disorder characterised by ataxia, spastic paraplegia, extrapyramidal dysfunction, cognitive deficits and optic atrophy, and it is almost exclusively seen in patients of Iraqi Jewish descent harbouring recessive *OPA3* mutations [10].

Charcot–Marie–Tooth (CMT) disease is one of the most common inherited neurological disorders, affecting approximately 1 in 2500 people in developed countries. A long list of causative genes have been identified, including *MFN2*, which can cause both recessive and dominant axonal CMT2A. In one particular subtype of CMT2A, referred to as hereditary motor and sensory neuropathy type 6 (HMSN6), dominant *MFN2* mutations result in visual failure secondary to optic atrophy in addition to the more typical axonal peripheral neuropathy [149, 150]. Prompted by this phenotypic association, other inherited forms of optic atrophy associated with CMT disease have been recently described with recessive mutations in *SLC25A46* [1] and *C12orf65* [107]. It should also be noted that both *SLC25A46* and *C12orf65* mutations can also result in a much more severe neurodegenerative Leigh-like presentation [11, 64, 69].

The Mohr-Tranebjaerg syndrome (deafness-dystonia-optic atrophy) is associated with recessive mutations affecting the *TIMM8A* gene on the X-chromosome, which encodes for a translocase of the mitochondrial inner membrane (TIMM) [70]. Visual failure in this disorder is thought to be due to global involvement of the visual pathways, extending as far back as the visual cortex [130]. A similar mechanism of altered protein import through the mitochondrial inner membrane has also been postulated for the DCMA (dilated cardiomyopathy with ataxia) syndrome, which is caused by mutations in the *DNAJC19* gene, and optic atrophy has been reported in some cases [45].

## Neurodegeneration: histopathological studies

**Fig. 4:**
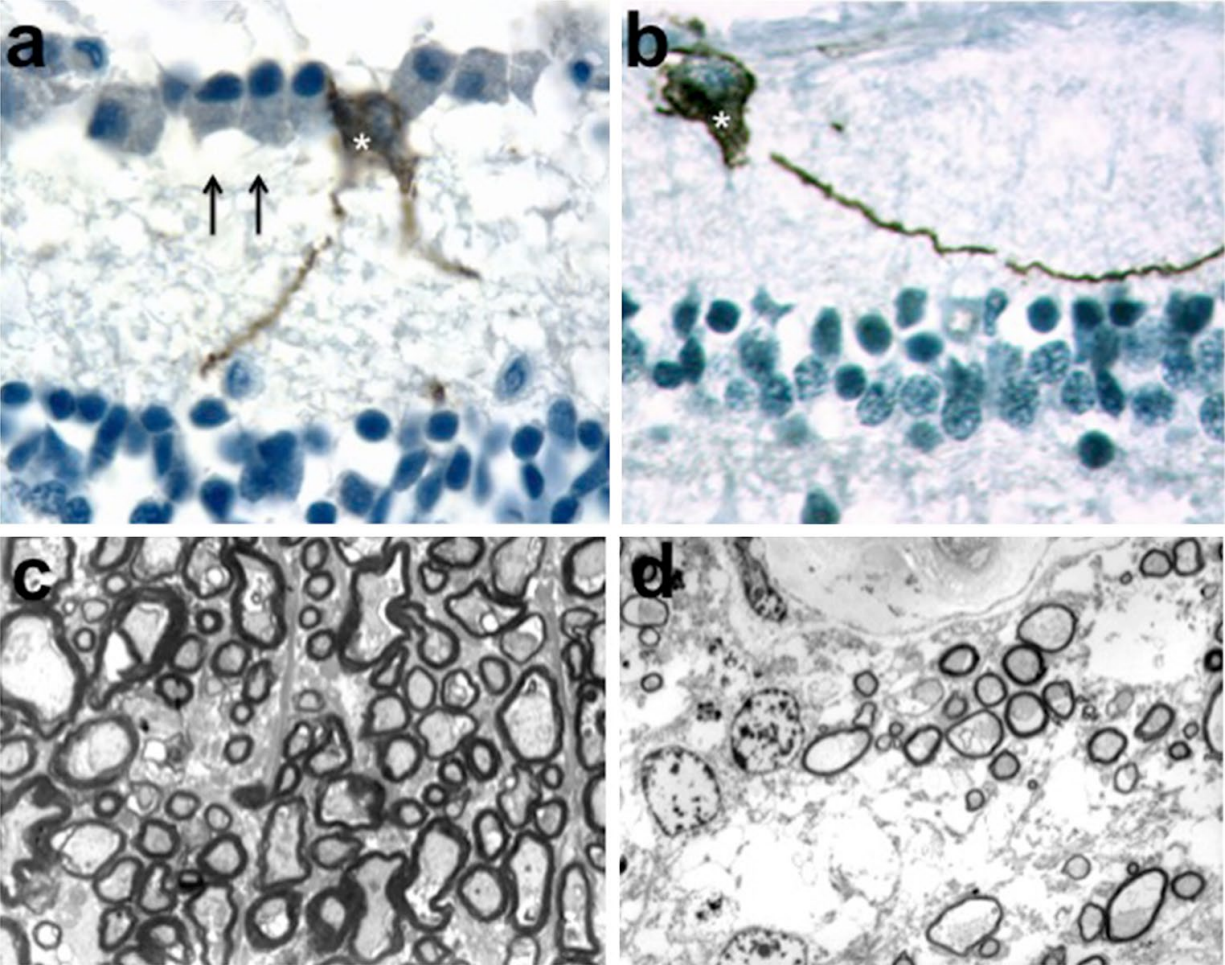
Melanopsin-expressing retinal ganglion cells and myelin ultrastructure. **a** Retinal cross-sections from a healthy control individual were stained with antibody against melanopsin. The inner retina shows the ganglion cell layer with retinal ganglion cells (*arrows*) and one melanopsin-expressing retinal ganglion cell (*asterisk*). **b** The inner retina from a patient with LHON reveals a melanopsin-expressing retinal ganglion cell (*asterisk*) in the complete absence of other retinal ganglion cells. **c** Electron micrograph of optic nerve cross-sections from a healthy control individual showing densely packed axons with variable axonal calibre and normal myelin thickness. **d** A representative illustration from a patient with LHON highlights the dramatic depletion of axons with a thin myelin coating around the surviving axons

The number of post-mortem studies for patients with mitochondrial optic neuropathies is limited and some historical reports date back to the pre-molecular era [2, 116]. For LHON, histopathological studies of multiple tissues including the retina, optic nerve and brain are rare and none have been performed during the acute stage of the disease [36]. Notwithstanding these practical limitations, there is severe loss of RGCs and marked thinning of the retinal nerve fibre layer (RNFL), but the remaining retinal structure is otherwise preserved [73, 119]. Electron microscopy carried out on optic nerve cross-sections has also highlighted prominent demyelinating features in the chronic stage of LHON with only a thin covering of myelin found around the remaining axons (Fig. 4) [36]. Comprehensive brain histopathology has been reported for an affected m.14484T>C LHON carrier with an MS overlap syndrome and a spectrum of neuropathological changes was observed, including demyelinating plaques within the brain white matter and optic nerves, and prominent vacuolation and cystic necrosis throughout the CNS [81]. As expected from the clinical features, post-mortem studies of optic nerves from patients with DOA confirmed the preferential loss of RGCs with evidence of axonal demyelination and gliosis [72, 77]. In contrast to the widespread loss of RGCs in patients with LHON and DOA, an evolutionary ancient RGC subtype that expresses the photopigment melanopsin (mRGCs) seems relatively immune to mitochondrial dysfunction and impaired mitochondrial dynamics. These mRGCs are highly specialised circadian photoreceptors that project to the hypothalamus and their preservation in LHON and DOA likely explains the maintenance of the pupillary light reflex even in those patients with severe visual impairment (Fig. 4) [82, 96].

## Neurodegeneration: in vivo imaging

Major advances in bioimaging technology have allowed unprecedented high-resolution images of the brain, visual pathways and retinal structures to be captured for analysis. In vivo techniques such as magnetic resonance spectroscopy (MRS) and optical coherence tomography (OCT) are non-invasive and well tolerated, and they provide standardised tools that have been used to document the chronology and pattern of tissue loss in both the acute and chronic stages of LHON [19, 21, 121]. Based on longitudinal OCT studies, swelling of the RGC axons along the inferotemporal segment of the optic disc seems to occur prior to the onset of visual loss and following disease conversion, a wave of RNFL swelling then spreads circumferentially to involve the remaining quadrants (Fig. 1) [16]. As optic atrophy ensues, there is global thinning of the peripapillary RNFL although some patients can exhibit relative sparing of the nasal quadrant in the chronic stage. High-resolution spectral domain macular OCT imaging has provided further insight into the pattern of RGC loss in LHON. Interestingly, in patients presenting with first eye involvement, the macular ganglion cell and inner plexiform layer (GC-IPL) and the macular RNFL already showed pathological thinning in the presymptomatic stage about 6 weeks before the onset of visual loss in the fellow eye. Maximal GC-IPL thinning was reached by 6 months and this OCT parameter could, therefore, prove to be a more sensitive biomarker of optic nerve damage compared with peripapillary RNFL measurements where progressive thinning continues until at least 12 months after the first onset of visual loss [14].

OCT studies in cohorts of patients harbouring pathogenic *OPA1* mutations have shown a differential pattern of RNFL thinning, which was more severe in the temporal quadrant and less pronounced in the nasal quadrant (Fig. 3) [20, 140]. Importantly, OPA1 mutations were associated with a significantly smaller optic nerve head and although speculative, this congenital hypoplasia could result from dysregulated apoptosis in early development and an accelerated loss of RGCs in utero. As for LHON, macular GC-IPL thickness measurements could prove a more useful structural biomarker that correlates better with visual acuity loss compared with peripapillary RNFL values [18]. Another observation made possible by high-resolution OCT imaging is the documentation of microcystic macular changes in the inner nuclear layer of patients with LHON and DOA [17, 29]. These retinal abnormalities are not associated with leakage on fluorescein angiography and they have been attributed to focal retinal schisis induced by vitreous traction on the background of severe RNFL atrophy. Similar microcystic macular changes have previously been described in patients with demyelinating optic neuritis and a possible aetiological link was made with the severity of the underlying inflammatory process [54]. More recently, lamination of the outer plexiform layer has been observed in patients with optic atrophy caused by dominant, but not recessive, *WFS1* mutations, and this peculiar OCT characteristic could be due to Müller cell dysfunction and disturbed calcium homoeostasis [86].

MRI tractography studies indicate that the loss of RGCs in the anterior visual pathway could result in transsynaptic degeneration that extends from the lateral geniculate nucleus to the optic radiations, as reported previously in other optic neuropathies such as demyelinating optic neuritis and glaucoma [22, 89]. More widespread microstructural white matter changes in mitochondrial optic neuropathies have also become more clearly apparent with the application of high-resolution MRI scanners and diffusion tensor (DT) imaging protocols to study cohorts of patients with LHON and OPA1-related DOA, which is consistent with the known white matter sensitivity to mitochondrial respiratory chain dysfunction [89, 94, 112, 114]. Furthermore, the pattern of DT MRI abnormalities and the lowered diffusivity in the white matter skeleton of the cerebellum, brainstem, thalamus and fronto-occipital-temporal lobes could reflect increased fragmentation of the mitochondrial network and altered neuronal dendritic arborization [112]. Proton magnetic resonance spectroscopy (^1^H-MRS) has also shown that raised lactate and reduced creatine (Cr) and N-acetylaspartate (NAA) levels can occur not only in areas of abnormal signal changes, but also in normal appearing white matter regions, indicative of a generalised mitochondrial metabolic deficit within the CNS [99, 114]. In terms of their clinical relevance, the neuroradiological changes observed in patients with mitochondrial optic neuropathies could prove useful surrogate biomarkers for the spectrum of white matter neuropathological abnormalities observed in mitochondrial disease, which can range from mild atrophy to extensive vacuolation with a sieve-like appearance depending on the severity of the underlying mitochondrial dysfunction [132].

## Disease mechanisms

The unique susceptibility of RGCs in mitochondrial optic neuropathies still remains a puzzling mystery and although several hypotheses have been put forward, none has been conclusively demonstrated [35, 143]. The highly specialised anatomical and physiological properties of RGCs are likely to be relevant, namely their relatively high metabolic requirements due to sustained spiking activity; the constant exposure to potentially damaging light; the sharp 90-degree bend as their axons exit the eye at the lamina cribosa; and the relatively long unmyelinated intraocular portion that necessitates a high density of packed mitochondria for efficient signal conduction [8, 27]. Faced with these multiple constraints, it should therefore not come as a surprise that mitochondria in neurones have evolved specific adaptations to survive in such a challenging environmental niche. The RGC body is located within the inner retina and its dense dendritic arborization together with its long axonal projection to the lateral geniculate nucleus places peculiar demands on cellular mitochondria. They must be able to cluster at synapses to deliver sufficient quantities of ATP locally and altering the delicate balance between mitochondrial fusion and fission is essential to meet the cell’s fluctuating bioenergetic demands, in addition to supporting bidirectional transport along dendrites and axon [31]. Clearly, the disruption of critical pro-fusion proteins, such as OPA1 and MFN2, or a direct impact on complex I subunits as in LHON will have profound negative consequences on mitochondrial function with reduced ATP production and elevated ROS levels [28]. There is still an ongoing debate whether the determining factor that drives RGC loss in mitochondrial optic neuropathies is primarily due to a bioenergetic crisis or toxic ROS levels, or possibly with both factors having a synergistic deleterious effect [83].

**Fig. 5:**
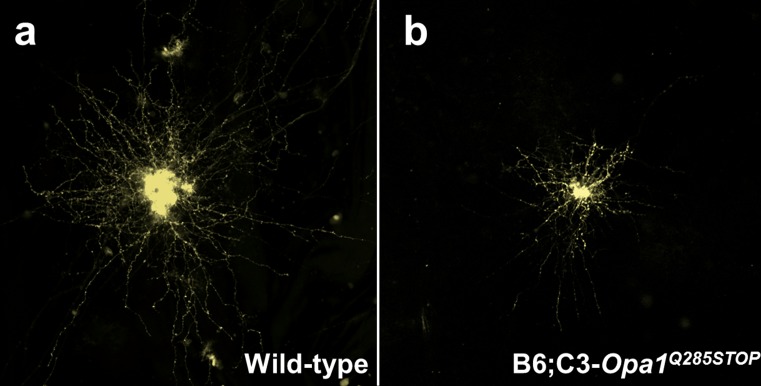
Dendropathy in a mouse model of dominant optic atrophy. **a** Flat-mounted retinas were labelled with the fluorescent marker DiI and individual RGCs were imaged with confocal microscopy. A representative RGC from a 16-month-old wild-type mouse is shown that demonstrates the extensive arborization of the dendritic tree. **b** An age-matched RGC from a B6;C3-*Opa1* ^Q285STOP^ mutant mouse harbouring a nonsense mutation in the *OPA1* gene. There is marked dendritic pruning characterised by a reduction in total dendritic length and field area

To further disentangle the complex neurodegenerative pathways that underpin this group of inherited optic nerve disorders, a number of in vitro approaches have been employed and the effort of several research groups worldwide has led to the generation of faithful animal models for both LHON and DOA [28, 84]. Heterozygous *Opa1* ^+*/*−^ mutant mice recapitulate the progressive RGC loss and optic nerve degeneration observed in the human form of the disease and the experimental data indicate that multiple pathways are involved that contribute to impaired mitochondrial oxidative phosphorylation, disturbed mitochondrial dynamics, increased ROS generation and, ultimately, a heightened susceptibility to undergo apoptosis [3, 40, 46, 120]. Immunolabelling of retinal flat-mount preparations obtained from *Opa1* ^+*/*−^ mutant mice showed dendritic pruning of ON-centre RGCs from 10 months of age with a significant reduction in total dendritic length and surface area (Fig. 5). Synaptic connectivity imaged after biolistic labelling of RGCs also showed reduced synaptic density on the distal ends of the dendritic tree [137]. Among the *Opa1* mouse models that have been established, there is also a suggestion that some of the mutant mice can develop extraocular features analogous to the more severe syndromic “plus” phenotypes observed in a proportion of patients with DOA. In addition to inner retinal and optic nerve degeneration, more widespread neuropathological and psychophysical abnormalities have been noted in subgroups of *Opa1* ^+*/*−^ mutant mice, including enlarged lateral ventricles within the brain, disorganised myofibres in skeletal and cardiac muscle, locomotive and ataxic gait disturbance, and decreased auditory brainstem responses [4, 40, 120].

Other cellular mechanisms that have gained increasing attention in the context of mitochondrial optic neuropathies are mtDNA maintenance and mitophagy, and the cross-talk with the endoplasmic reticulum at MAM interfaces [28, 129]. The accumulation of somatic mtDNA deletions in post-mitotic tissues of patients carrying *OPA1* mutations points towards an impaired mtDNA replication machinery and there is a suggestion that OPA1 could be part of the physical structure that anchors nucleoids to the mitochondrial inner membrane [48]. Another possible explanation for the clonal expansion of somatic mtDNA deletions is the inability to remove these dysfunctional mtDNA species through mitophagy [34, 102]. The occurrence of optic atrophy in the context of pathogenic *MFN2*, *WFS1* and *CISD2* mutations provides strong supportive evidence that RGC survival is detrimentally affected as a result of impaired interactions between the mitochondrial network and the endoplasmic reticulum, especially if there is disturbed calcium flux between these compartments [28]. Although much has been learnt over the years, all the above observations still do not fully explain the marked clinical variability and tissue specificity seen in mitochondrial optic neuropathies, and dissecting these complex mechanistic questions holds the key to more effective targeted therapeutic strategies [141]. Rightly so, the early focus has been on the molecular genetics, but now there is an urgent need to capitalise on the greater availability of more sophisticated technological platforms to compare the transcriptomic, proteomic and metabolomic profiles between different phenotypic groups to look for potential clues or biomarkers that might explain the variable disease penetrance and prognosis for patients with mitochondrial optic neuropathies [44, 131].

## Therapeutic strategies

The management of patients with mitochondrial optic neuropathies remains largely supportive with the provision of visual rehabilitation and genetic counselling [106, 148]. There are a number of technical and logistical challenges in developing effective disease-modifying treatments aimed at rescuing RGCs, namely the need to bypass the blood brain barrier to achieve a high enough local concentration of the drug; the limited therapeutic window posed by the massive early loss of RGCs in subacute disorders such as LHON; the limited availability of sensitive optic nerve biomarkers; and, crucially, the substantial financial resources needed to conduct an adequately powered treatment trial. Despite these difficulties, the eye is an easily accessible organ for direct surgical intervention and it benefits from relative immune privilege.

One obvious therapeutic approach is to improve the flux of high energy electrons along the mitochondrial respiratory chain and bypass any blockage in more proximal complexes. Ubiquinone is a fat-soluble molecule present at a very high concentration within the inner mitochondrial membrane and it has the advantageous property of transferring electrons efficiently from complexes I and II to complex III [60]. Co-enzyme Q10 (CoQ_10_) is a synthetic ubiquinone analogue and despite the limited evidence base, it is frequently prescribed to patients with mitochondrial disease. Idebenone and EPI-743 are newer generation shorter-chain analogues of ubiquinone and, unlike CoQ_10_, they can cross the blood brain barrier, which makes them theoretically more potent as antioxidants [106]. There is supporting evidence that idebenone can improve the visual prognosis, albeit, partially, in a subgroup of patients that are treated relatively early in the acute stage of LHON [33, 79, 80]. EPI-743 has also shown promise in a small case series of five patients with acute LHON treated within 90 days of disease conversion, but a randomised placebo-controlled trial is needed to fully explore its true therapeutic potential [117]. In addition to antioxidants, boosting mitochondrial biogenesis and ATP synthesis is an attractive complementary strategy that could help rescue failing neuronal cells from apoptotic cell death. A number of drugs, such as bezafibrate, rosiglitazone, 5-aminoimidazole-4-carboxamide ribonucleoside (AICAR), acetyl-L-carnitine (ALCAR), vitamin B3, and oestrogen-like molecules, have been identified as effective inducers of mitochondrial proliferation under conditions of metabolic stress and their effectiveness in rescuing the optic nerve phenotype or the neurological manifestations of LHON and DOA deserve to be studied further [106, 148].

Direct mitochondrial gene therapy is challenging due to the constraints imposed by the organelle’s physical structure and the presence of a relatively impervious inner mitochondrial membrane. A possible approach to circumvent these technical difficulties is allotopic gene expression where the gene of interest is transferred into the nuclear genome and the replacement protein product is engineered with a specific targeting sequence that facilitates its uptake into the mitochondrial compartment [139]. Promising pre-clinical data based on in vitro and rodent models have resulted in the recent launch of pivotal clinical trials for patients harbouring the m.11778G>A mutation that involves the intravitreal injection of a modified adeno-associated virus (AAV) vector carrying the replacement *MTND4* subunit gene (NCT02652767, NCT02652780 and NCT02161380, https://clinicaltrials.gov/ct2/home, accessed on 28 August 2016) [49, 61]. An alternative, but complementary, approach is to modify the AAV construct to carry a gene whose protein product has a neuroprotective effect and enhances the survival of dysfunctional neurones. Proof of principle has been demonstrated with the overexpression of *SOD2*, which encodes for superoxide dismutase and serves to boost the cell’s antioxidant defence mechanisms [108]. These more generic neuroprotective approaches are attractive as they could be applied not only to rescue RGCs, but also other central neuronal populations in patients manifesting the more generalised “plus” phenotypes. There are still multiple challenges that need to be overcome to achieve a higher efficiency of RGC transfection and sustained transgene expression, but gene therapy programmes for mitochondrial optic neuropathies caused by dominant *OPA1* and recessive *WFS1* mutations are currently in development, which will hopefully lead to early phase clinical trials.

The coming decade will bring together a confluence of major advances in cell-based and genomic editing technologies that hold the potential to revolutionise our approach to the prevention and treatment of human genetic diseases [139]. The ability to reprogram post-mitotic somatic cells into induced pluripotent stem cells (iPSCs) has been a major breakthrough and the ability to produce patient-specific neuronal populations, including RGCs, opens up not only the possibility of personalised regenerative medicine, but in the shorter term, high-throughput drug screening on the diseased cells of interest can be actively exploited. Although the RGC layer is easily accessible, multisystem neurological involvement represents a major challenge and another strand of research has focused on the prevention of disease transmission from mother to child. There is an estimated 2300 women of childbearing age in the United Kingdom that harbour pathogenic mtDNA mutations and by using the national fertility rate, about 150 newborn per year would be at high risk of developing severe mitochondrial disease [56]. A pioneering therapeutic approach is to use conventional IVF techniques to transfer the parental nuclear genetic material into a donor egg containing a normal wild-type mtDNA population such that the child’s entire genetic make-up will be derived from the biological parents except for the 37 mitochondrial genes. Pronuclear transfer and metaphase II spindle transfer are the two techniques that are currently being refined and further experimental work will hopefully validate the safety of these potentially groundbreaking mitochondrial replacement strategies [43, 67, 127, 128]. Following a wide-ranging public consultation, in February 2015, both Houses of Parliament in the United Kingdom have voted strongly in favour of mitochondrial donation to prevent the maternal transmission of mitochondrial disease (http://www.wellcome.ac.uk/About-us/Policy/Spotlight-issues/Mitochondrial-diseases/, accessed on 28 August 2016). Safety must remain the primary concern at all times and tight regulations must be agreed and implemented at national and international levels to avoid misuse of these promising, but as yet unproven, methods aimed at preventing the maternal transmission of pathogenic mtDNA mutations to the next generation.

## Conclusions

All the causative genes identified so far in patients with inherited optic neuropathies result in mitochondrial dysfunction and it is abundantly clear that RGCs are exquisitely sensitive to even minor disturbances in mitochondrial biogenesis or dynamics, with an innate susceptibility to undergo apoptosis under conditions of heightened cellular stress and elevated ROS levels. It is still difficult to fully unravel the individual contribution of these often overlapping pathophysiological mechanisms but, nevertheless, they do provide a number of targets amenable to therapeutic modulation. The pace of gene discovery will continue to accelerate with the advent of next-generation sequencing technologies, providing not only a confirmed molecular diagnosis to patients and their families, but also important insights into new protein mediators that regular RGC and neuronal survival. The stark reality is that the majority of patients with mitochondrial optic neuropathies are registered legally blind and the translational gap for this group of disorders remains to be bridged. However, all the signs point towards a turning point and the availability of better disease models coupled with innovation in bioimaging, drug discovery and genetic manipulation brings renewed hope that clinicians will soon be in a position to better monitor disease progression and improve the visual prognosis of patients with mitochondrial optic neuropathies.

## Electronic supplementary material

Below is the link to the electronic supplementary material.

Supplementary material 1 (DOC 631 kb)
