# Supplementary material for: A neurodegenerative perspective on mitochondrial optic neuropathies
Source: Acta Neuropathol. 2016 Sep 30;132(6):789–806. doi: 10.1007/s00401-016-1625-2 (PMC5106504; doi:10.1007/s00401-016-1625-2)
Supplement: Supplementary file 1 — Supplementary material 1 (DOC 631 kb) [file 401_2016_1625_MOESM1_ESM.doc]

## Supplementary Appendix

Optical coherence tomography (OCT) is a non-invasive imaging modality that allows high-resolution cross-sectional images of the retina to be obtained at different anatomical locations. There has been a rapid refinement of the technology and most softwares allow automated segmentation and reliable measurements of the various retinal layers (**Supplementary Figure 1**). A number of commercial platforms are in routine clinical use, but as they do not provide comparable data, the same platform should be used for longitudinal comparison of a patient or to standardize data collection for a specific study (<https://commondataelements.ninds.nih.gov/Doc/Mito/F1885_Vision_Mitochondria_Disease_OCT_Guidelines.doc>, accessed on 8 August 2016). Two commonly used OCT imaging protocols are the macular cube (512x128), which is centred on the fovea, and the optic disc cube (200x200), which is centred on the optic disc (**Supplementary Figure 2**). The analysis software automatically selects the appropriate normative age range for the subject and the measurements are represented within colour-coded distribution centiles: (i) red < 1%, (ii) yellow 1-5%, and (iii) green 5-95%.

### Supplementary Figure 1. Segmentation of Individual Retinal Layers.


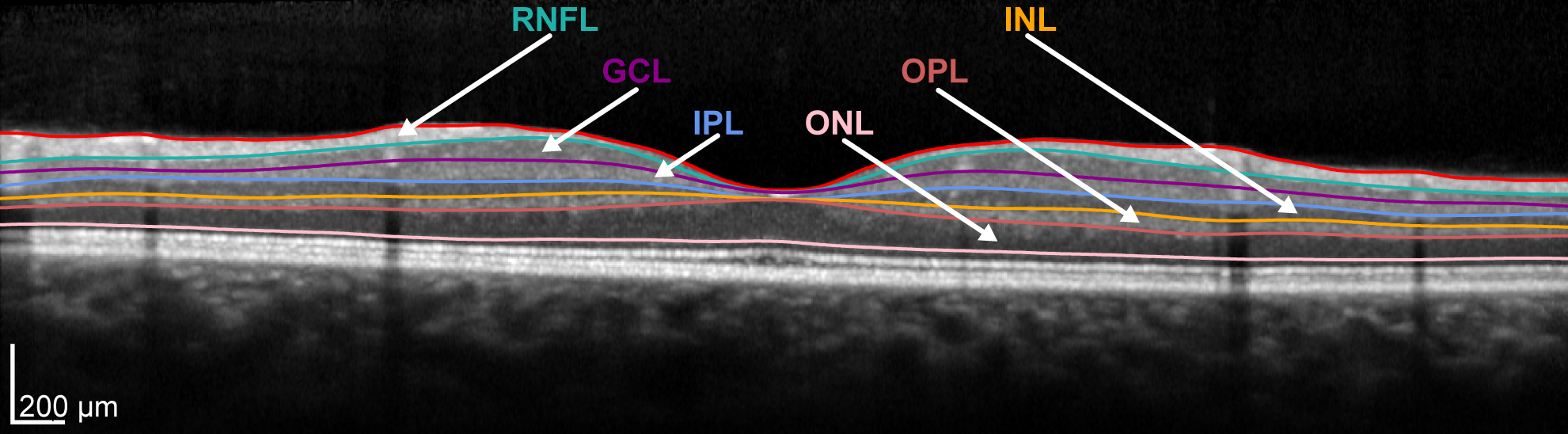


Ganglion cell layer (GCL); inner nuclear layer (INL); inner plexiform layer (IPL), outer nuclear layer (ONL); outer plexiform layer (OPL); retinal nerve fibre layer (RNFL). (<https://openi.nlm.nih.gov/detailedresult.php?img=PMC4562656_pone.0137316.g002&req=4>, accessed on 28 August 2016)

### Supplementary Figure 2. Macular and Optic Disc Cube Measurements.


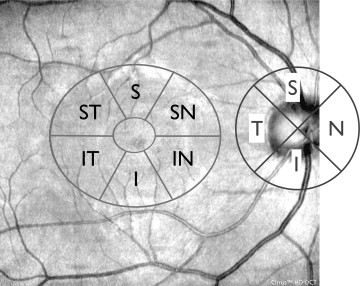


The anatomical locations of the macular cube (512x128) and optic disc cube (200x200) protocols have been illustrated for the spectral-domain Cirrus^TM^ platform (Carl Zeiss Meditec, Dublin, CA). The macular cube provides a measurement of the retinal ganglion cell and inner plexiform layer (GC-IPL) thickness at the macula, which is measured from an elliptical annulus centred on the fovea. The thickness measurements (in µm) are provided: (i) as an average; (ii) as a minimum; and (iii) for 6 individual sectors: superotemporal (ST), superior (S), superonasal (SN), inferonasal (IN), inferior (I), and inferotemporal (IT). The optic disc cube measures the retinal nerve fibre layer (RNFL) thickness from a circle that is 3.46 mm diameter wide and centred around the optic disc. The thickness measurements (in µm) are provided: (i) as an average; (ii) for the temporal (T), superior (S), nasal (N), and inferior (I) quadrants, and (iii) as individual clock hours.
